# Matrix eigenvalue solver based on reconfigurable photonic neural network

**DOI:** 10.1515/nanoph-2022-0109

**Published:** 2022-04-25

**Authors:** Kun Liao, Chentong Li, Tianxiang Dai, Chuyu Zhong, Hongtao Lin, Xiaoyong Hu, Qihuang Gong

**Affiliations:** State Key Laboratory for Mesoscopic Physics & Department of Physics, Collaborative Innovation Center of Quantum Matter, Beijing Academy of Quantum Information Sciences, Nano-optoelectronics Frontier Center of Ministry of Education, Peking University, Beijing 100871, China; College of Information Science & Electronic Engineering, Zhejiang University, Hangzhou 310027, China; Collaborative Innovation Center of Extreme Optics, Shanxi University, Taiyuan, Shanxi 030006, China; Peking University Yangtze Delta Institute of Optoelectronics, Nantong, Jiangsu 226010, China

**Keywords:** graphene/Si thermo-optical modulation, matrix eigenvalue solver, reconfigurable photonic neural network, saturated absorption effect

## Abstract

The solution of matrix eigenvalues has always been a research hotspot in the field of modern numerical analysis, which has important value in practical application of engineering technology and scientific research. Despite the fact that currently existing algorithms for solving the eigenvalues of matrices are well-developed to try to satisfy both in terms of computational accuracy and efficiency, few of them have been able to be realized on photonic platform. The photonic neural network not only has strong judgment in solving inference tasks due to the superior learning ability, but also makes full use of the advantages of photonic computing with ultrahigh speed and ultralow energy consumption. Here, we propose a strategy of an eigenvalue solver for real-value symmetric matrices based on reconfigurable photonic neural networks. The strategy shows the feasibility of solving the eigenvalues of real-value symmetric matrices of *n* × *n* matrices with locally connected networks. Experimentally, we demonstrate the task of solving the eigenvalues of 2 × 2, 3 × 3, and 4 × 4 real-value symmetric matrices based on graphene/Si thermo-optical modulated reconfigurable photonic neural networks with saturated absorption nonlinear activation layer. The theoretically predicted test set accuracy of the 2 × 2 matrices is 93.6% with the measured accuracy of 78.8% in the experiment by the standard defined for simplicity of comparison. This work not only provides a feasible solution for the on-chip integrated photonic realization of eigenvalue solving of real-value symmetric matrices, but also lays the foundation for a new generation of intelligent on-chip integrated all-optical computing.

## 1 Introduction

The eigenvalue problem of matrix is an important content in matrix theory [1], [2], [3]. The solution of matrix eigenvalues has always been a research hotspot in modern numerical analysis. Many problems in engineering technology and scientific research can usually be attributed to solving the eigenvalues of a matrix and its corresponding eigenvectors, such as image compression in computer vision [4, 5], vibration problems [6] and determination of some critical values in physical systems [7], stability of dynamic systems [8], and some mathematical modeling problems [9]. Therefore, the solution of matrix eigenvalues is of great value in practical application [10, 11]. At present, the numerical algorithms for solving the eigenvalues of matrices can be divided into decomposition methods [12, 13] and iterative methods [14, 15]. The decomposition method decomposes the original matrix into a form that is easier to find the eigenvalues. The iterative method calculates the eigenvalue as the limit of an infinite sequence. These methods have their own disadvantages in terms of efficiency or accuracy. More importantly, none of these methods have been realized in integrated photonic platform. The existing algorithms for solving the eigenvalues of the matrix for optical problems are difficult to meet the requirements of ultrahigh speed and ultralow energy consumption computing. Therefore, there is no effective method to effectively solve the eigenvalues of different types of matrices on photonics platforms. As one of the artificial intelligence algorithms, neural network has strong judgment in solving inference tasks because of its superior learning ability [16, 17]. In addition, neural networks implemented on photonic platforms can take advantages of photonic computing, including ultralow energy consumption [18, 19], ultra-fast time response [20, 21], low integration crosstalk [22], [23], [24], and multidimensional degrees of freedom [25, 26]. In recent years, photonic neural networks have achieved superior performance in artificial intelligence tasks such as pattern recognition [27] and image classification [28].

Here, we propose a strategy of eigenvalue solver for real-value symmetric matrix based on reconfigurable photonic neural network. The strategy shows the feasibility of using locally connected photonic neural networks to solve the eigenvalues of real-value symmetric matrices with different orders. In experiments, we use graphene/Si thermo-optical modulated reconfigurable photonic neural networks with saturated absorption nonlinear activation layer to demonstrate the task of solving the eigenvalues of 2 × 2, 3 × 3, and 4 × 4 real-value symmetric matrices. After the training process, the accuracy of the test set is 93.6%, and the accuracy of the test set measured in the corresponding experiment is 78.8%. This work not only provides a feasible solution for the realization of on-chip integrated photonics for solving real-value symmetric matrix eigenvalues, but also further expands the function of photonic neural network, laying the foundation for the new generation of intelligent on-chip integrated all-optical computing.

## 2 Results and discussion

### 2.1 Strategy of matrix eigenvalue solver based on photonic neural network

#### 2.1.1 The framework of the proposed photonic neural network

**Figure 1:**
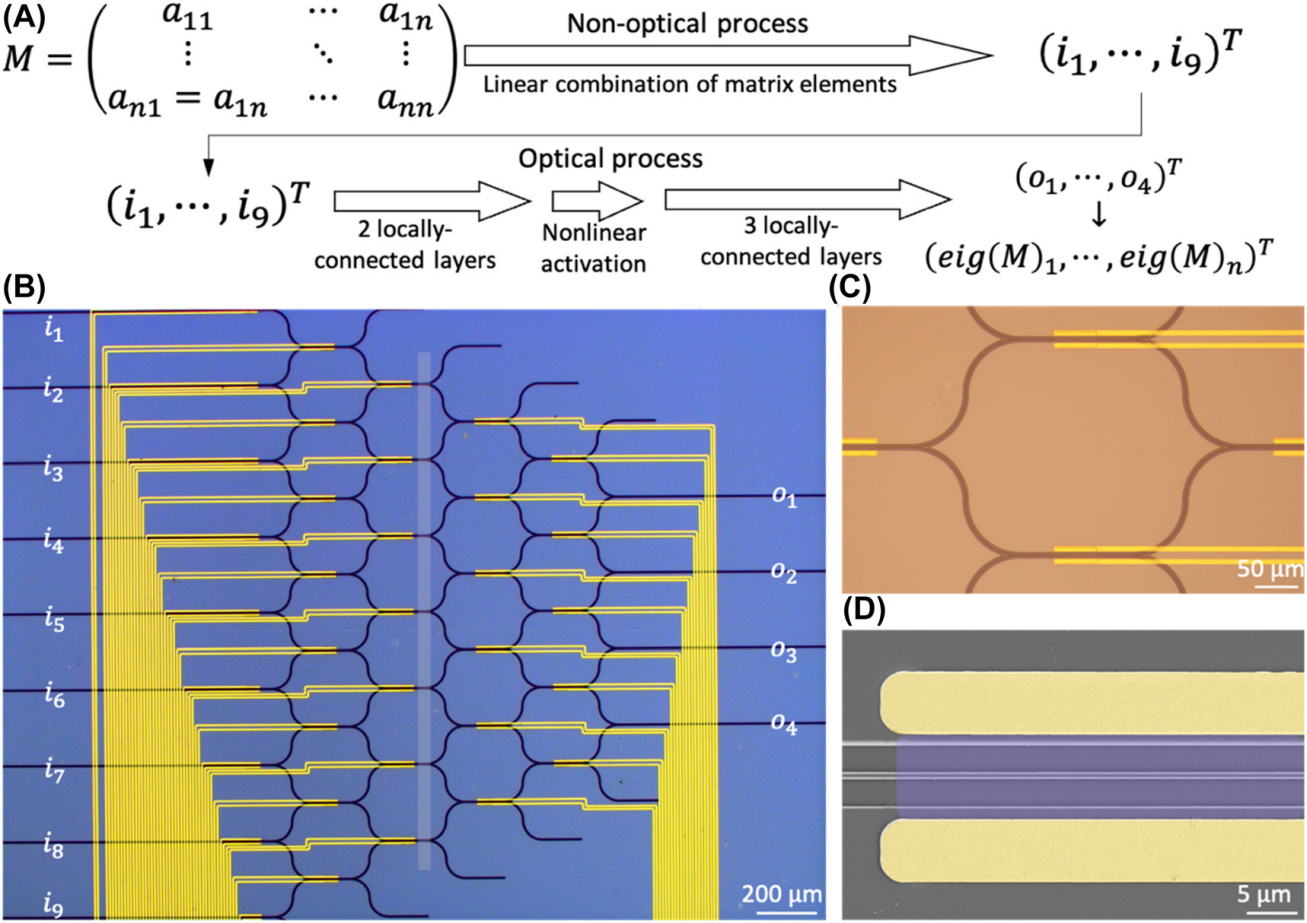
The framework of the proposed photonic neural network. (A) Schematic diagram of the process for realizing the target task. Elements of the symmetric matrix are first linearly combined into input information for the optical part of the network. Signals goes through locally-connected layers and nonlinear layers and are coupled out to represent eigenvalues of the original matrix. (B) Optical micrograph of the characteristic structure of the proposed network with 9 input ports (*i*_1_–*i*_9_) and 4 output ports (*o*_1_–*o*_4_). (C) Optical micrograph of one single cell which contains two phase shifters and a merging structure. (D) Electron micrograph of a phase shifter for thermo-optical modulation, purple: single-layer graphene; yellow: Au/Cr electrodes.

The problem we aim to solve with the photonic neural network we propose is finding the eigenvalues of symmetric matrices for they are widely encountered in physical problems (Figure 1A). To begin with, we consider solving the eigenvalue problem for 2 × 2 symmetric matrices with non-negative real-value elements and eigenvalues. Additionally, we restrict the elements in the matrices between 0 and 10. This restriction here will not limit the performance of the network since any other matrices can be obtained through linear stretching with one matrix in the restricted domain. The proposed network is also designed to solve the eigenvalue problem of *n* × *n* matrices with similar conditions.

The architecture of the photonic neural network comprises one linear fully-connected layer and a five-layer locally-connected structure with nine input ports and four output ports (Figure 1B). The five-layer structure has a nonlinear activation of the form:

(1) $$T=1-\left(\alpha_{\text{NS}}+\frac{\alpha_{\text{S}}}{1+I/I_{\text{s}}}\right)$$

where *α*_S_, *α*_NS_, and *I*_s_ are values of material feature layer placed behind the second locally-connected layer. Here, we encoded the input and output value with light intensity. Information of the elements in a matrix would be presented by the intensity of light in the input ports of the optical part of the network (*i*_1_–*i*_9_). The network then gives an array of light intensity in the output ports (*o*_1_–*o*_4_), which represent the predicted eigenvalue of the matrix. To focus on the vital part of the problem, the linear fully-connected layer is performed in non-optical ways, although we noticed several optical ways to perform such an operation had been established [29].

The first layer of the five-layer structure has eight neurons, each has a shared phase shifter with its neighboring unit (Figure 1C), and the next layer has seven neurons and the successive layer has one fewer, resulting in 35 tunable weights in total. In addition, we introduced two additional weights to be trained. The first is a factor of the input light intensity, i.e., the ratio of intensity, since the nonlinear activation function works differently with different intensity. The other is a factor of output ratio, which linearly scale the relationship between output intensity and the eigenvalue it stands for, i.e., the ratio of output. This factor is considered because unlike the electronic neural network, optical layers cannot change the intensity of light in a free and direct way. Therefore, the absolute value of the output signal may not fit the scale in the dataset.

The nonlinear layer is chosen to present different level of transparency when light of different intensity attempts to pass through it. The form of transparency function for a typical saturated absorber is chosen to be in the form of Eq. (1) above [30]. In term of physical meaning, *α*_S_ and *α*_NS_ are the saturable and nonsaturable absorption, *I*_s_ is the saturation intensity, defined as the optical intensity required in a steady state to reduce the absorption to half of its unbleached value. Additionally, we noticed that the operation of interference is not linear in the real-value domain represented by intensity, but is linear in the complex-value domain represented by complex amplitude. Therefore, if we look from the complex-value perspective of the neural network, the operation of calculating the intensity naturally present another nonlinear activation after the final layer.

A *n* × *n* symmetric matrix has *n*(*n* + 1)/2 independent elements. Our strategy to acquire training sample is to randomly generate these elements and form matrices that satisfy the restrictions above. Eigenvalues of the matrices are obtained through traditional computational methods with NumPy and are ranked from big to small. The generated elements and calculated eigenvalues (targets) form a labeled dataset for training. For each training process, a labeled training set contains more than 2000 samples. The training of the neural network follows a standard process. In one epoch of the training process, the input data is forward-propagated and compared with the target, returning a loss by the loss function of the following form:

(2) $$\text{LOSS}=\underset{i}{\sum }{\left(R_{o}\times I_{o_{i}}-T_{i}\right)}^{2}$$

where *R*_o_ is output ratio, *I*_o*i*_ is the output intensity at port *i*, and *T*_*i*_ represents the target value. Then the loss of all input set is combined and backward-propagated to obtain the gradient of each weight. Then optimization process is performed by the optimizer Adam [31]. The whole process is done with PyTorch toolbox.

2000 test sets for each *n* × *n* matrices are generated in the same way with the training set. During the generation process, data that overlap with the training dataset is picked out so that no data leakage happens during the training and testing process. We determine that one prediction by the neural network is accurate if the deviation between each element of the prediction and its corresponding label is lower than 1 and the sum of the two deviations is lower than 1.2 for the 2 × 2 matrix eigenvalue solver, which express as:

(3) $$\left\{ \begin{array}{c} \max\left(\left|R_{o}\times I_{o_{i}}-T_{i}\right|\right)<1\\ \sum \left(\left|R_{o}\times I_{o_{i}}-T_{i}\right|\right)<1.2 \end{array}\right.$$

This standard of accuracy takes into account both individual performance of predicting each eigenvalue and these performances combined.

#### 2.1.2 The 2 × 2 matrix eigenvalue solver

**Figure 2:**
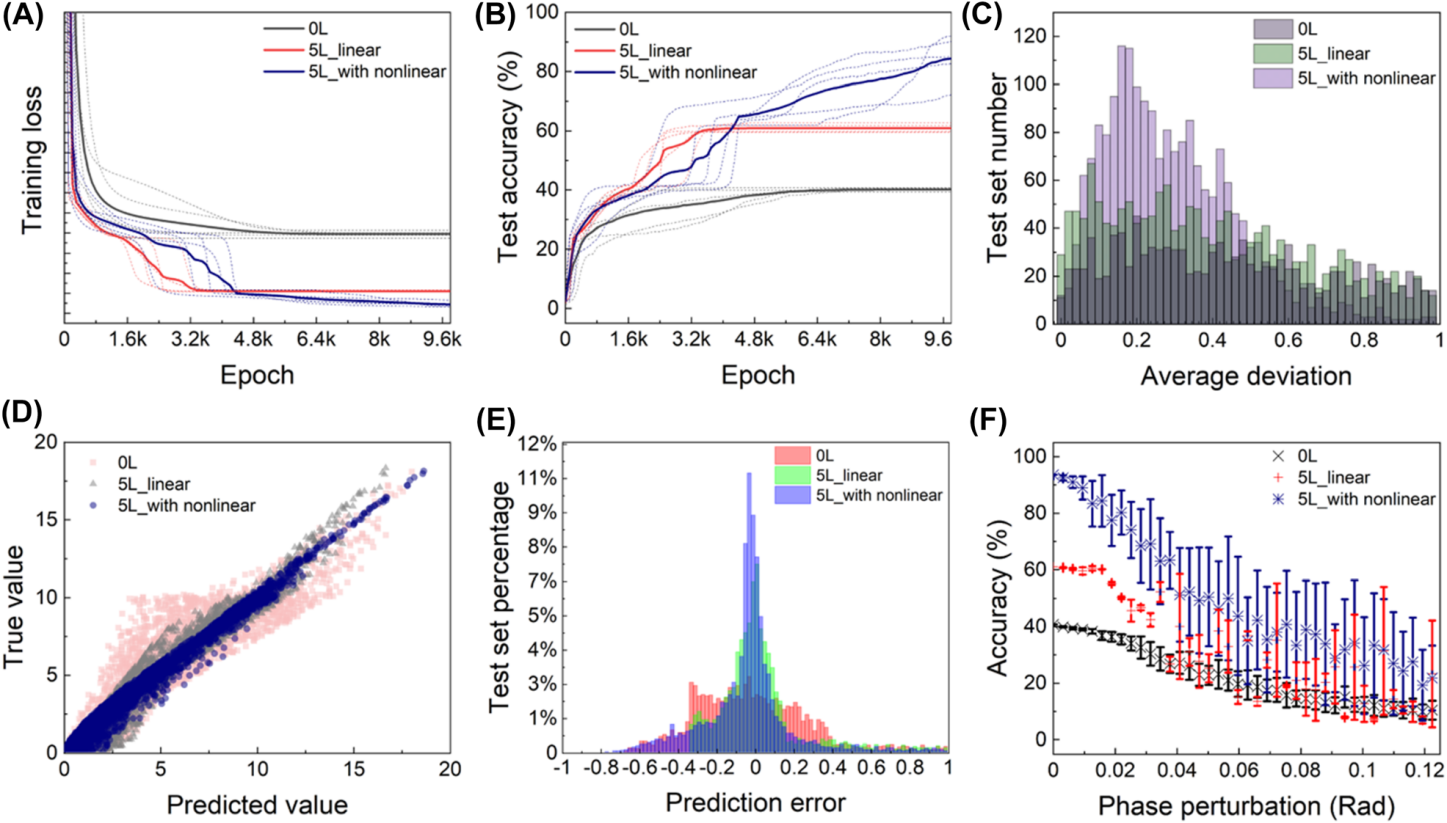
Performance of the 2 × 2 matrix eigenvalue solver. (A) Training loss reduction comparison among linear combination (0L), linear combination plus 5-layer structure (5L) and linear combination plus 5-layer structure with nonlinear activation function (5L with nonlinear). Solid lines represent average loss of 5 training attempts depicted by dotted lines. (B) Comparison of test set accuracy among the three configurations above, where solid lines represent average loss of 5 training attempts depicted by dotted lines. (C) Distribution of the average test deviation of the three configurations above, which is cut off after the deviation of 1. (D) Correlation between the true value and the predicted value for 0L, 5L, and 5L with nonlinear condition. (E) Relative deviation for 0L, 5L, and 5L with nonlinear condition. (F) Test accuracy reduction of the three configurations above under random phase perturbation on phase shifters. Error bar represents standard variance of 20 random attempts.

To illustrate the function of our proposed photonic neural network and show that the optical part does play an important role, we performed training and testing on three network configurations to realize the 2 × 2 matrix eigenvalue solver. One with only a linear fully-connected layer which contains no optical process (labeled with 0L), another with a linear fully-connected layer and 5 optical locally-connected layers as illustrated above (labeled with 5L), and finally, one with a linear fully-connected, 5 optical locally-connected layers and nonlinear activation function in the form above (labeled with 5L with nonlinear). In the 2 × 2 problem, after 5 times repeating training per 10000 epochs, the training loss and test set accuracy are shown in Figure 2A and B. Obviously, the 5L with nonlinear configuration has the least training loss and the highest test accuracy with the 5L configuration in the second place. Considering the standard above, we have achieved 40.8% of test accuracy for 0L configuration, 61.0% for 5L configuration and more than 93.6% for the 5L with nonlinear configuration. In order to show the difference of the three configurations more clearly, in Figure 2C we illustrated the distribution of test set numbers against average deviation, which is defined as the average of absolute distance between each output element and their corresponding target, namely:

(4) $$d_{\text{avg}}=\frac{\sum \left|(R_{o}\times I_{o_{i}}-T_{i}\right|}{D}$$

where *D* is the order of the problem. It is obvious that the average deviation distribution of 5L with nonlinear configuration is much denser near 0, and the 5L layer is also better than 0L. Also investigated are the correlation between the true value and the predicted value (Figure 2D), and the relative deviation defined by $\frac{R_{o}\times I_{o}-T}{T}$ for each individual eigenvalue prediction (Figure 2E). These results suggest that the proposed network has to some degree addressed a solution to the eigenvalue problem for 2 × 2 symmetric matrices. In order to deal with random errors in experiments, we looked into the robustness of the network. Here, we added random noise to the trained weight to see if the accuracy maintains. For the 2 × 2 problem, as shown in Figure 2F, up to 0.01 rad of random phase error would not affect the performance of the network much. Accuracy drops quickly after 0.02 rad of error. It is noticed that the optical layer and the nonlinear layer result in increased instability of the network, which is a natural result for more complicated structure. The error analysis here provides a direction and scope for dynamic reconfigurable modulation in the experiment.

#### 2.1.3 The 3 × 3 and 4 × 4 matrix eigenvalue solver

To generalize our result to higher order condition, we performed the same process in 3 × 3 and 4 × 4 conditions. In order to emphasize on the function of the proposed neural network, we made a few adjustments to the training and testing process. The input range is further limited from real numbers between 0 and 10 to non-negative integers up to 5. The standard of accuracy is also changed to individual deviation lower than 1, removing the total deviation limitation. This standard shows the possibility of one prediction to fall within a distance of 1 of the targets.

**Figure 3:**
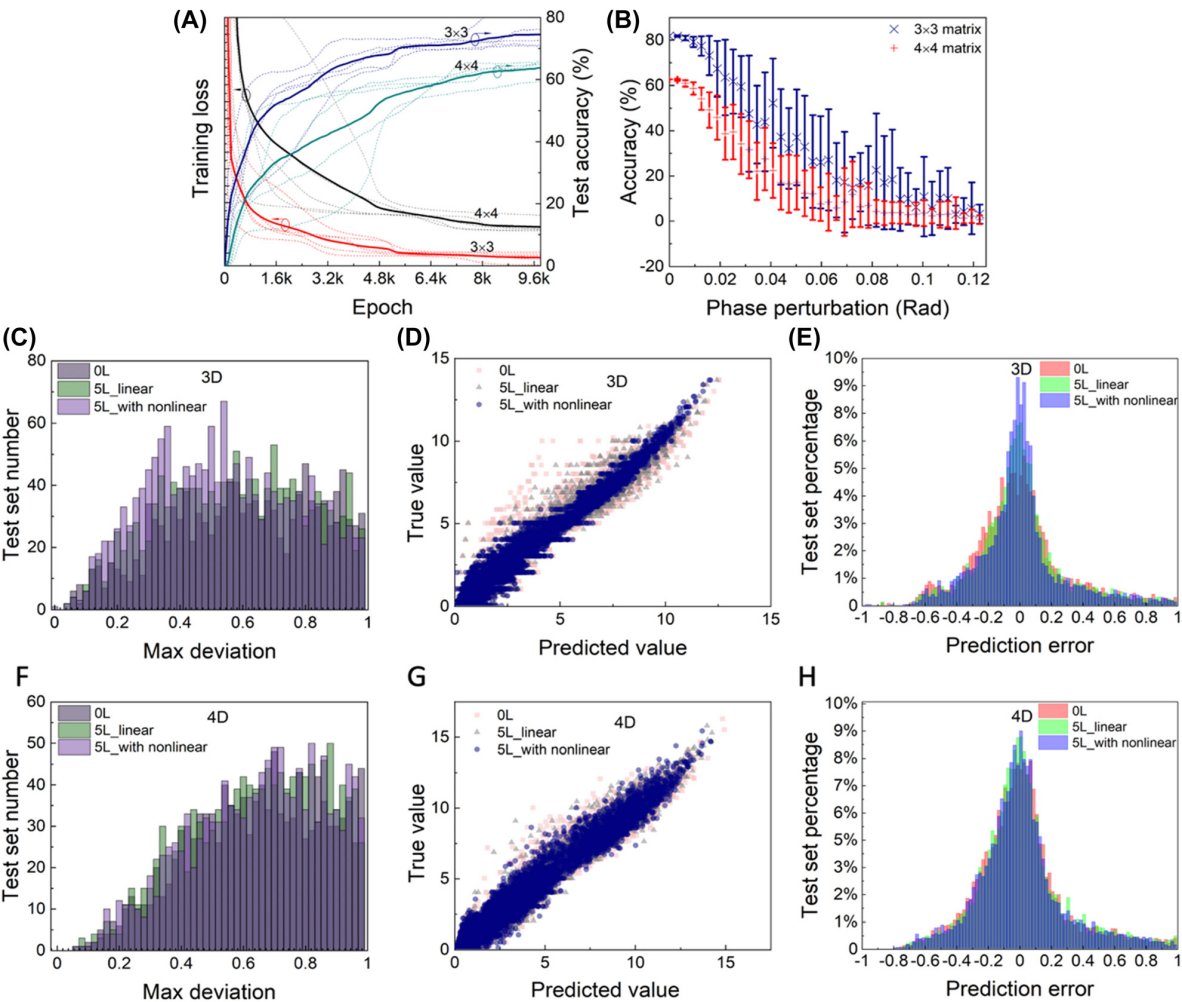
Performance of higher order matrix eigenvalue solvers. (A) Training loss and test accuracy versus training epoch of the 3 × 3 and 4 × 4 problems with the configuration of 5L with nonlinear. Solid lines represent average loss of 5 training attempts depicted by dotted lines. (B) Test accuracy reduction of the 3 × 3 and 4 × 4 problems under random phase perturbation on phase shifters. Error bar represents standard variance of 20 random attempts. (C, F) Distribution of the max deviation of the 3 × 3 and 4 × 4 problem, respectively, which are cut off after the deviation of 1. (D, G) Correlation between the true value and the predicted value for 0L, 5L, and 5L with nonlinear condition for 3 × 3 and 4 × 4, respectively. (E, H) Relative deviation for 0L, 5L, and 5L with nonlinear condition for 3 × 3 and 4 × 4, respectively.

The training and testing process of 3 × 3 and 4 × 4 problem is similar with the 2 × 2 one but with different datasets and criteria. We performed training and testing process to the 0L, 5L, and 5L with nonlinear configurations, the loss descent and accuracy ascent of the 5L with nonlinear configurations of 3 × 3 and 4 × 4 problem is shown in Figure 3A. The stability against perturbation of phase is shown in Figure 3B. Also, the distribution against max deviation, which is defined by the max distance between one output elements and the corresponding target value for each matrix, namely:

(5) $$d_{\text{max}}=\frac{\text{max}\left(\left|R_{o}\times I_{o_{i}}-T_{i}\right|\right)}{D}$$

are illustrated in Figure 3C and F. As observed from the figure that in the 3 × 3 condition the three configurations still distinguish from each other with the 5L with nonlinear one has the best performance. But in higher order problems the three configurations are mostly overlapping with each other. This might be a result from insufficient training since it may need larger computational parameter space. Also, the test accuracy becomes lower for *n* × *n* matrices. The calculated test set accuracy of 2 × 2, 3 × 3, and 4 × 4 is summarized in Table 1. Besides, the same correlation relation and relative deviation are investigated as 2 × 2 for 3 × 3 and 4 × 4 condition in Figure 3D, E, G and H.

**Table 1:** Comparison of the theoretical calculated and experimental measured test set accuracy of the eigenvalues for different order matrices.

	2 × 2 (strict)		3 × 3		4 × 4	
	Theoretical	Measured	Theoretical	Measured	Theoretical	Measured
0L	40.8%	\	61.3%	\	58.3%	\
5L linear	61.0%	49.2%	72.0%	67.1%	65.2%	62.2%
5L with nonlinear	93.6%	78.8%	82.3%	73.5%	63.9%	57.6%

### 2.2 Reconfigurable photonic neural network for matrix eigenvalue solver

We realized the thermo-optical modulated reconfigurable photonic neural networks based on silicon photonic chip fabricated by micro-nano processing technology. First, we used electron beam lithography (EBL) with an exposure precision of 10 nm (JEOL) combined with dry etching technology of inductively coupled plasma (ICP) (OXFORD PlasmaPro 100 Cobra 180) to fabricate a cascaded silicon waveguide network. Then, the single-layer graphene was transferred on the surface of the waveguide structure. The transferred single-layer graphene was covered at the specific position as the saturable absorption layer by using the UV photolithography as a mask (Suss MA/MB6) and the RIE etching (OXFORD PlasmaPro 100 RIE). Afterwards, we used plasma-enhanced chemical vapor deposition (PECVD) to deposit 150 nm-thick SiO_2_ (OXFORD PlasmaPro 100 PECVD) as a cladding layer on the waveguide structure, and then transferred a large area of single-layer graphene covering the waveguide structure on the cladding layer. We then used UV photolithography and RIE etching to make the transferred single-layer graphene on the cladding layer function as thermo-optical modulators at corresponding positions. Finally, we used the UV photolithography and the lift-off technology to make electron beam evaporated (KURT J.LESKER Labline PVD 75) Cr/Au 10 nm/100 nm as the metal electrodes for thermo-optical modulation. A sample of a silicon-based photonic neural network chip supporting thermo-optical modulation was obtained from the above steps.

**Figure 4:**
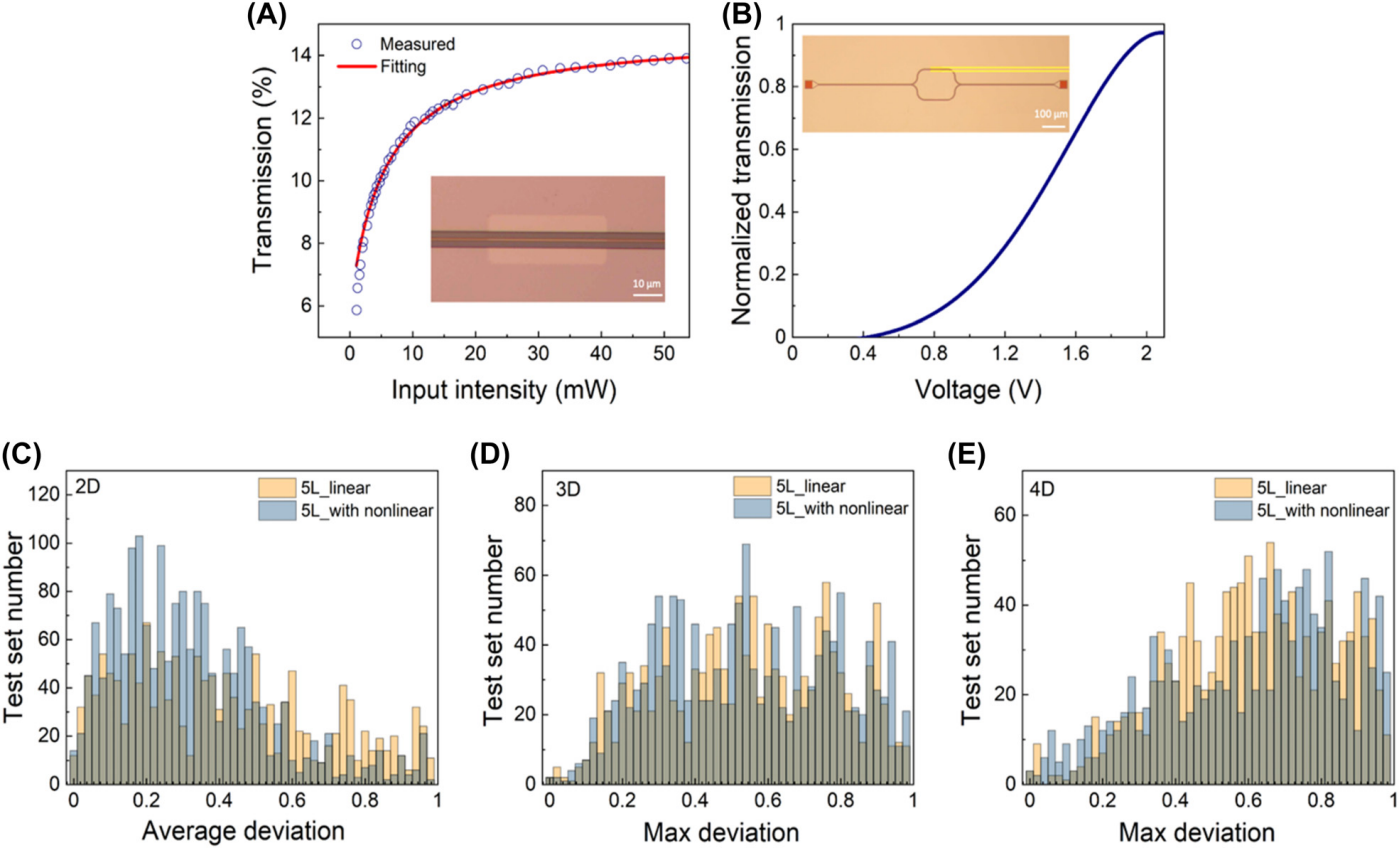
Reconfigurable photonic neural network for matrix eigenvalue solver. (A) The optical nonlinear activation layer of single-layer graphene covered on the waveguide with the length of 40 μm. The home-built femtosecond pulse fiber laser system (central wavelength: 1560 nm, pulse width: 80 fs) is used as the light source. The fitting parameters are *α*_S_ = 0.088 *α*_NS_ = 0.852 *I*_s_ = 5.446. Inset: the optical micrograph of optical nonlinear activation cell. (B) The change curve of normalized light intensity transmission with modulation voltage in the test interference structure of optical waveguide with *V*_*π*_ = 2.11 V and *P*_*π*_ = 10.2 mW. Inset: the optical micrograph of thermal-optical modulated test interference structure of optical waveguide. (C–E) Distributions of the experimentally measured test set with deviation of eigenvalues of different order matrices for 5L and 5L with nonlinear, with the 2 × 2 corresponding to the average deviation, 3 × 3 and 4 × 4 corresponding to the maximum deviation.

The optical nonlinear saturated absorption effect of single-layer graphene covered on the waveguide at the specified position is used as the nonlinear activation layer with the fitting parameters *α*_S_ = 0.088, *α*_NS_ = 0.852, *I*_s_ = 5.446 (Figure 4A). In this experiment, the length of single-layer graphene as the saturated absorption layer is 40 μm (inset of Figure 4A). And we realized the thermo-optical modulated reconfigurable photonic neural network by using a single-layer graphene with a length of 50 μm on a 150 nm-thick SiO_2_ cladding layer (Figure 1D). Figure 4B shows the change curve of normalized light intensity transmission with modulation voltage in the test interference structure of optical waveguide. Here we realized the modulation of the optical phase from 0 to *π* with the voltage of 2.11 V and the power of 10 mW. The voltage control resolution of the used multi-channel ultra-precision driver (Qontrol systems, Q8iv) can reach 180 μV, with the maximum output current (per channel) of 24 mA and the power supply range of 12 V–30 V. Here, 7 drivers (8 channels each) are used to modulate the total of 53 phase shifters (modulation of 35 wt + modulation of 9 × 2 input channels) in the proposed networks.

Here, we demonstrated the solution of eigenvalues of 2 × 2, 3 × 3, and 4 × 4 matrices for a 5 optical locally-connected layers and a 5 optical locally-connected layers with nonlinear activation layers on an optical fiber array coupled system. The test set for each order is 2000. The distribution of the experimentally measured test set numbers with deviation is shown in Figures 4C–E, with the 2 × 2 corresponding to the average deviation, 3 × 3 and 4 × 4 corresponding to the maximum deviation. It can be seen from the 2 × 2 test set distribution that the performance of the photonic neural network with the single-layer graphene as the saturated absorption layer is better (accuracy 78.8%) than that with only linear layer (accuracy 49.2%). The total computing time can be considered as characterized by the time-of-flight of light through the entire structure (including the non-characteristic structure including input-output waveguide and coupling ports) is about 90 ps, which is much faster compared with ∼10^−4^ s calculated on electronic computer with NumPy (Intel Core I7 2.6 GHz processor). Besides, the integrated photonic platform can also claim ultralow energy consumption. The computation energy overhead is estimated as hundreds of fJ/bit based on the laser pulse power we used in our experiments. Therefore, the proposed strategy of eigenvalue solver based on the integrated reconfigurable photonic neural network features ultrafast and energy-efficiency computation compared with other strategy implemented on nonphotonic platforms. As the order of the matrix to be solved increases, the advantage of a network with nonlinear activation layers decreases, because the size of the parameter space of the network becomes the dominant factor as the complexity of the problem increases. The degree of freedom in our demonstrated structure becomes insufficient for *n* × *n* matrices and more complex tasks. We also summarize the comparison of the theoretical calculated and experimental measured accuracy of the eigenvalues of different order matrices (Table 1). The consistency between the experimental results and the calculated results shows that the designed eigenvalue solver based on the reconfigurable photonic neural network is indeed realized in experiment.

We noticed that the difference between the theoretical and the experimental accuracy was affected by the error of fabrication, variation of phase shifters and the performance of the saturated absorption material in the system. A supplement gradient descent training with the fabricated system, where the gradient is approximated by finite difference, can be helpful for addressing the problem and restoring the device to the optimal condition.

### 2.3 Scalability of the proposed strategy for the matrix eigenvalue solver

The requirement for experimental demonstration limits our network to 5 layers as presented above, but our design can be scaled up to adapt to different input sizes, as well as increasing depth and parameter number for the same input size. More importantly, a larger network with enough channels will eliminate the need for a non-optical linear combination process, enabling a full optical neural network structure.

Our scalable structure consists of two types of blocks: reduction blocks and padded blocks. Reduction blocks share the same structural pattern as our experimental design, which becomes narrower with every layer. A reduction block with *n* input channels and *m* output channels (*n* > *m*) contains *n* − *m* layers, a total of $\frac{1}{2}\left(n+m-1\right)\left(n-m\right)$ phase parameters. Padded blocks, on the other hand, does not reduce the number of channels. A padded block consists of locally connected layers with padding 0 and 1 that appears in turn. We define one padded block with *n* input channels to contain *n* layers, where *n* is an even integer, which has a total of $n^{2}-\frac{1}{2}$ phase parameters. This number of layers in padding blocks is set to *n* because it takes exactly *n* layers to provide connectivity for all channels in the network. A nonlinear layer is appended to the final layer of every block.

For a much deeper network, the energy decay of every layer leads to the intensity value falls out of the sweet spot for the nonlinear layer. In addition to applying nonlinear layers to every block instead of every layer, we also introduce a gaining layer for every 8 layers that brings average intensity of the layers back to 1. The gain parameter of the layer, marked as *β*, updates with every forward iteration:

(6) $$x_{\text{out}}≔x_{\text{in}}/\beta_{n}$$

(7) $$\beta_{n+1}≔\alpha \beta_{n}+\left(1-\alpha \text{Mean}\left(\text{Abs}\left(x_{\text{in}}\right)\right)\right)$$

By scaling down *α* over the training process, the parameter *β* converges, resulting in an optimal gain value, which can be applied in future experiments.

The output of our network is measured in intensity values, which has limited range. But the eigenvalues of matrices cannot be assumed to fall in the same range. In order to scale the output value of the network, we introduce an additional scaling factor *γ* to the last layer of the network:

(8) Output=γ×Input

This factor, unlike the gaining factor *β*, is not updated independently, but is trained with the network. This final scaling factor would be part of the detector in experiment.

Thus, it is possible to scale up the network by introducing padded blocks inside the original network structure. For example, our original network can be viewed as a reduction block with 9 input channels and 4 output channels. By replacing the fourth layer with a padded block, the resulting network consists of one reduction block with 9 input channels and 6 output channels, one padded block with 6 layers, and one reduction block with 6 input channels and 4 output channels, with a total of 11 layers. This process can be repeated many times to build a much deeper network with additional parameters.

**Figure 5:**
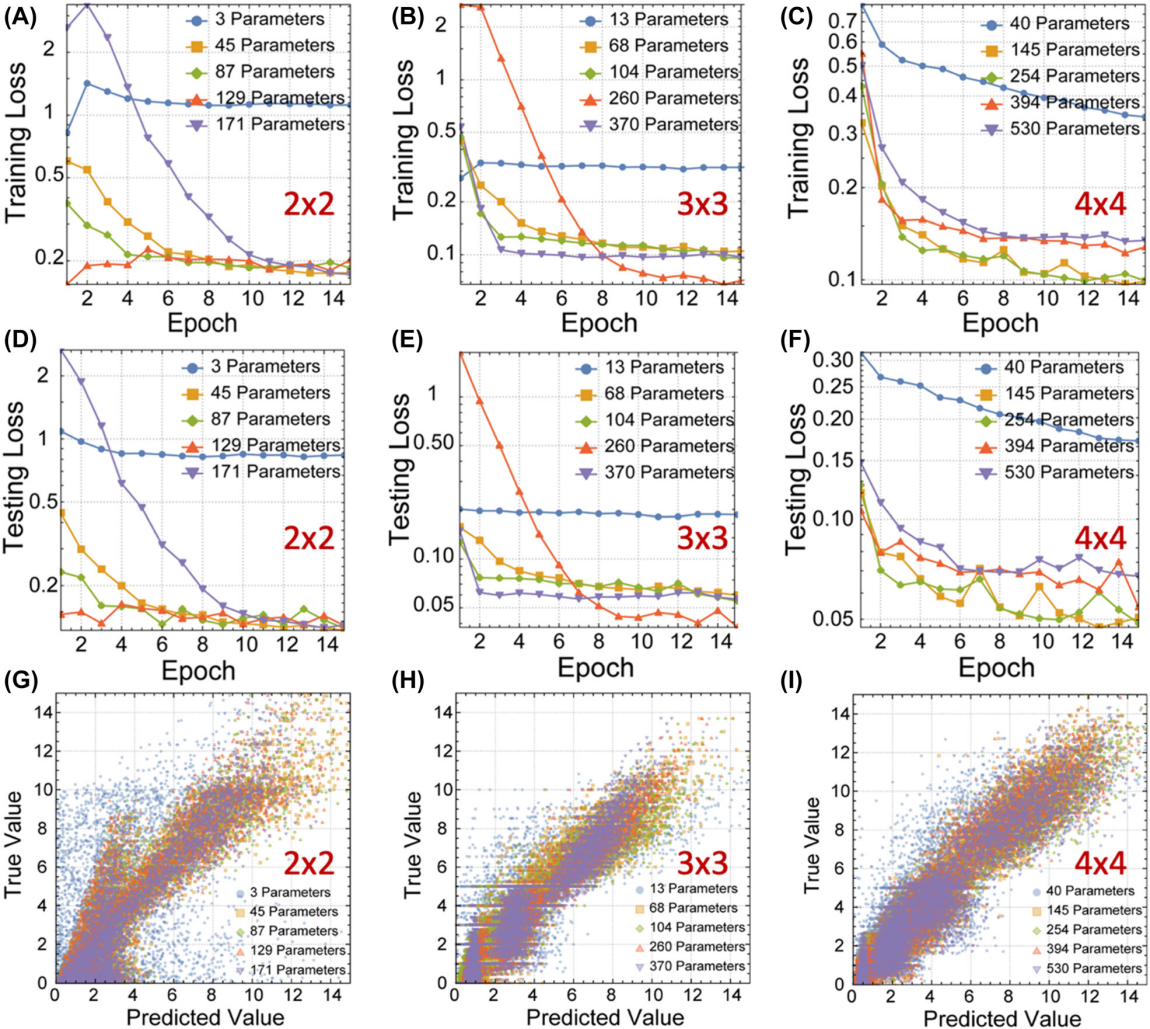
Training and testing results of upscaled eigenvalue solver for 2 × 2, 3 × 3, and 4 × 4 matrices. (A–C) The training loss for 2 × 2, 3 × 3, and 4 × 4 matrices eigenvalue solver for each parameter condition with inserted padded blocks. (D–F) The testing loss for 2 × 2, 3 × 3, and 4 × 4 matrices eigenvalue solver for each parameter condition with inserted padded blocks. (G–I). The correlation diagram of true value and predicted value of 2 × 2, 3 × 3, and 4 × 4 matrices eigenvalue solver for each parameter condition with inserted padded blocks.

Starting from a pure reduction block with 3/6/10 input channels and 2/3/4 output channels for matrices of size 2/3/4, we present 4 scaled-up versions of the network with 1/2/3/4 additional padded blocks. The additional padded blocks are evenly spaced. These networks with the gaining layers and scaling factors introduced are trained on the same dataset also using Adam optimizer from the same PyTorch platform. Each of the networks is trained with a minibatch size of 16 for 15 rounds. The results are shown in Figure 5. It can be seen from the figure that both training and testing losses decrease with parameter numbers, up to an extent, and increases with too much parameters due to overfitting. The predicted eigenvalues are also much closer to the actual values.

To demonstrate that this structural pattern applies to larger inputs, we generated a 8 × 8 matrix dataset with 100,000 samples, using the first 75,000 as training set and the last 25,000 as testing set. The matrix elements are no longer limited to be positive, following the normal distribution with a standard deviation of 10. The matrix elements are directly used as the input value for the network, which contains 64 input channels and 8 output channels.

**Figure 6:**
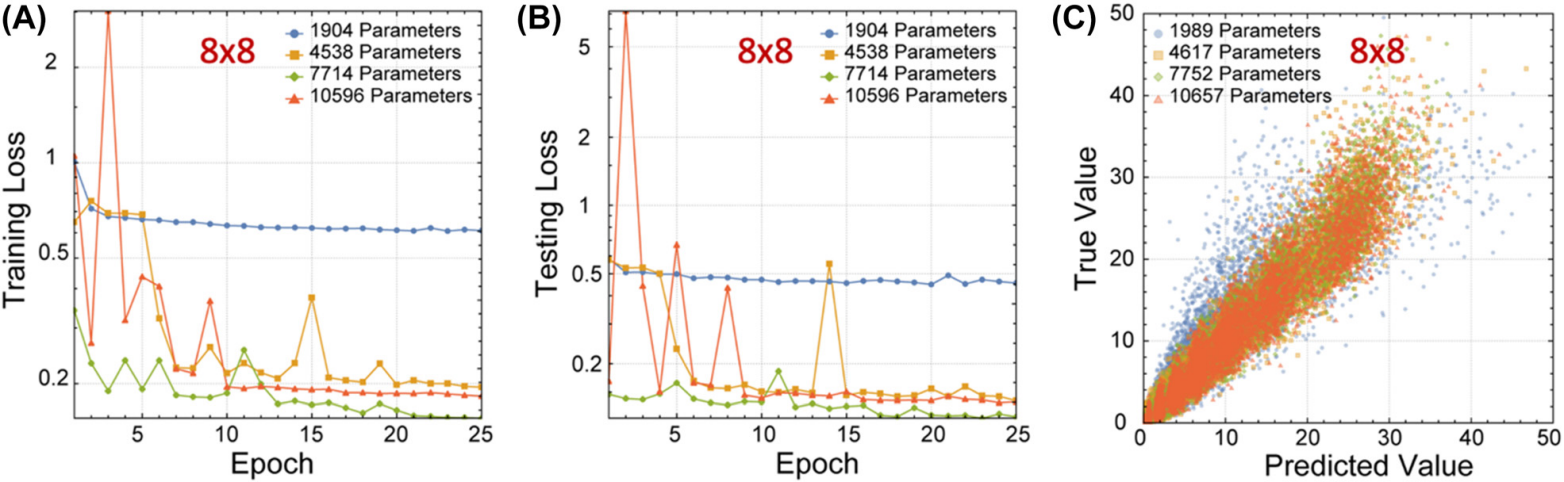
Training and testing results of upscaled eigenvalue solver for 8 × 8 matrices. (A) The training loss for 8 × 8 matrices eigenvalue solver for each parameter condition. (B) The testing loss for 8 × 8 matrices eigenvalue solver for each parameter condition. (C) The correlation between true value and predicted value for 8 × 8 matrices eigenvalue solver for each parameter condition.

This larger network, starting from a reduction block with 56 channels, is scaled up in the same way, to a total number of 10596 parameters. The 4 networks are trained for 25 rounds, using a minibatch size of 32 with the same optimizer on the larger dataset. The results are shown in Figure 6. Our network achieves decent accuracy on this dataset, but the resulting accuracy drops for having more parameters. This is due to the network being too deep, as the network with 10596 parameters contains 271 locally connected layers, which is much higher than the normal depth of CNNs without skip connections. It is shown [32] that the key component in successfully training networks with hundreds of layers is by introducing skip connections to the network structure. However, our network structure does not support skip connections in hardware, due to the waveguide’s inability to cross over. One possible solution for this is to introduce weighed skip connections at initialization, while gradually decreasing the skip connection weight to zero during the training process. It is also possible to keep the skip connection, but to merge it with network weights after training to build the final network. These solutions will be further explored in the future.

## 3 Conclusions

In order to provide an integrated photonic method to effectively solve the eigenvalues of different types of matrices with ultrahigh speed and ultralow energy consumption, here, we proposed a strategy of eigenvalue solver of real-value symmetric matrix based on reconfigurable photonic neural network. We experimentally realized the graphene/Si thermo-optical modulated reconfigurable photonic neural network with saturated absorption nonlinear activation layer to demonstrate the task of solving the eigenvalues of 2 × 2, 3 × 3, and 4 × 4 symmetric matrices with moderate accuracy. This work expands the function of photonic neural network, laying the foundation for the new generation of intelligent on-chip integrated all-optical computing.
